# Supplementary material for: Content-rich biological network constructed by mining PubMed abstracts
Source: BMC Bioinformatics. 2004 Oct 8;5:147. doi: 10.1186/1471-2105-5-147 (PMC528731; doi:10.1186/1471-2105-5-147)
Supplement: Additional File 2 — The original results of the above study (non-essential files are deleted to keep the file size under the limit set by BMC bioinformatics). [file 1471-2105-5-147-S2.bz2 › chilibotAdditionalFile2/dip10/445ID9468473E10451/html/CCL4_CCR5.html]

 


 **CCL4** and **CCR5** 
  
Found 58 abstracts in PubMed, retrieved 10.  
 

 What does Google say? 
 PDF only 
| .edu only 

---

**Interactive relationship** (e.g. stimulation, inhibition, etc)

**Neutral relationship**- However, the remaining expression of CCR1 and  **CCR5**  correlated reciprocally with an ongoing unresponsiveness to the CCR1 and  **CCR5**  agonists CCL3 macrophage inflammatory protein 1alpha MIP 1alpha,  **CCL4**  MIP 1beta, and CCL5 regulated on activation, normal T expressed and secreted RANTES, all chemokines binding to these two receptors.  Ref: 12885942 J Leukoc Biol, 2003
- High levels of CCL3 and  **CCL4**  were produced within 8 h after infection, which is likely to account for the observed  **CCR5**  down modulation on Mtb infected DC.  Ref: 12538673 J Immunol, 2003

**Non-interactive relationship** (e.g. studied together, co-existance, homology, etc.)

- We examined the responses of human peripheral blood T cells, present in an ECM like context, to combinatorial signaling transduced by SDF 1alpha CXCL12, and two  **CCR5**  ligands, RANTES CCL5 and MIP 1beta  **CCL4** .  Ref: 12502723 Int Immunol, 2003
- Characterization of the role of the N loop of MIP 1 beta  [ **CCL4** ]  in  **CCR5**  binding.  Ref: 12427015 Biochemistry, 2002
- After 2 to 4 weeks, L. amazonensis infected mice had significantly delayed and depressed expression of inflammatory cytokines interleukin 12 IL 12, gamma interferon, IL 1alpha, IL 1beta, CC chemokines CC chemokine ligand 3 CCL3 macrophage inflammatory protein 1alpha MIP 1alpha,  **CCL4**  MIP 1beta, CCL5 RANTES, MIP 2, and chemokine receptors CCR1, CCR2,  **CCR5**  in foot tissues and draining lymph nodes compared to the expression in L. major infected controls.  Ref: 12874303 Infect Immun, 2003
- In LAG  [ **CCL4** ]  3 matured DC, surface expression of  **CCR5**  was down regulated and CCR7 was up regulated.  Ref: 12547595 Vaccine, 2003
